# Differential diagnosis of left buttock pain in an 87-year-old woman: a clinical vignette

**DOI:** 10.12701/jyms.2026.43.19

**Published:** 2026-02-16

**Authors:** Ji Hwan Kim, Mathieu Boudier-Revéret, Soo Hyun Ahn, Min Cheol Chang

**Affiliations:** 1 Department of Physical Medicine and Rehabilitation, Yeungnam University College of Medicine, Korea; 2 Department of Physical Medicine and Rehabilitation, Centre Hospitalier de l'Université de Montréal, Montreal, QC, Canada

## Patient information

An 87-year-old woman presented with left buttock pain of approximately 1 month in duration. She had a medical history of hypertension and multiple osteoporotic vertebral body compression fractures at T11, L2, L3, and L5. A bone mineral density test, performed 2 years ago, confirmed osteoporosis with a T-score of −3.1, and she had been receiving subcutaneous injections of denosumab (60 mg) at 6-month intervals [1]. On initial evaluation, she reported severe pain rated 8/10 on the numeric rating scale, which impaired her daily activities and ambulation. The pain was exacerbated by standing or walking and alleviated by lying down. She reported no identifiable precipitating events such as falls or injuries. The patient did not report fever or weight loss. Physical examination revealed intact motor strength and sensation in both lower extremities, with symmetrically decreased deep tendon reflexes in the knees and ankles. No objective neurological deficits were observed. In addition, there was no tenderness over the lumbar facet joints or interspinous areas, or any significant tenderness over the gluteal musculature. However, both Patrick’s (flexion, abduction, and external rotation [FABER]) test and the sacroiliac joint (SIJ) thrust test were positive on the left side, reproducing her left buttock pain.

## Diagnostic assessment

**Fig. 1.**
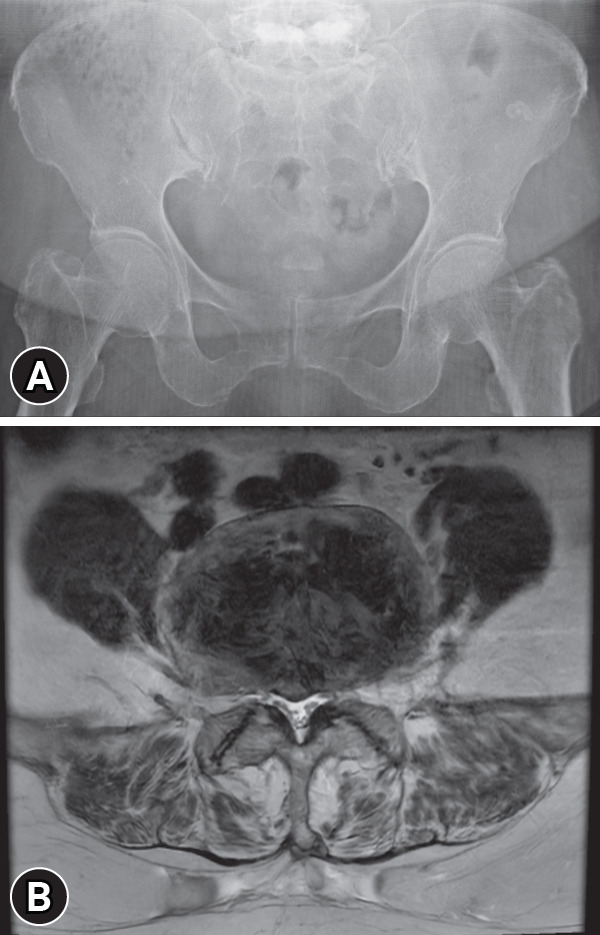
(A) Pelvic radiograph showing degenerative changes in the bilateral sacroiliac joints. (B) Axial T2-weighted lumbar magnetic resonance imaging demonstrating central canal stenosis at L4 to L5.

Pelvic anteroposterior radiography revealed an irregularity in the SIJ space, with subchondral sclerosis on both sides (Fig. 1A). Lumbar spine magnetic resonance imaging (MRI) revealed chronic compression fractures of L2, L3, and L5, and central stenosis at the L4 to L5 level (Fig. 1B).

### 1. Differential diagnosis

#### 1) L5 radicular pain

Lumbar radicular pain was considered a leading possibility because lumbar MRI demonstrated central canal stenosis at L4 to L5, which can lead to left L5 radicular pain. Furthermore, the pain was aggravated by standing and walking, which aligned with pain characteristics typically observed in patients with spinal stenosis. Spinal stenosis is one of the most common disorders that cause buttock pain [2]. Therefore, left L5 radicular pain due to spinal stenosis at L4 to L5 was considered the most likely cause of our patient’s symptoms.

#### 2) Sacroiliac joint-origin pain

SIJ-origin pain often results from degenerative arthropathy, mechanical dysfunction, or inflammation of the SIJ and surrounding ligaments, and is often elicited by specific provocative maneuvers. Pelvic radiography revealed bilateral SIJ degeneration. Additionally, positive findings in Patrick’s FABER test and the SIJ thrust test supported the possibility that our patient’s left buttock pain was caused by SIJ degeneration [3].

#### 3) Sacral insufficiency fracture

Sacral insufficiency fracture (SIF) occurs when physiological loading exceeds the strength of osteoporotic bone, most commonly involving the sacral alae, and presents with severe buttock or pelvic pain without antecedent trauma [4,5]. Given the patient’s history of severe osteoporosis and pain aggravated by standing or walking, SIF was considered. Moreover, sacral fractures could have induced a positive response to SIJ provocative maneuvers. Although pelvic radiography did not show evidence of a fracture, SIF could not be ruled out because MRI or computed tomography is often necessary for a definitive diagnosis.

#### 4) Facet-origin pain

Facet-origin pain arises from degenerative or inflammatory changes in the facet joints, which can cause lower back pain and possibly referred pain in the buttocks [6]. However, tenderness was not noted over the lumbar facet joints, and the patient experienced left buttock pain without lower back pain, making facet joint pain less likely. Nevertheless, because tenderness assessments can yield false negative results and the distinction between buttock pain and lower back pain can be challenging in clinical practice, facet joint pain could not be completely excluded [7].

#### 5) Discogenic pain

Discogenic pain is thought to result from degenerative disc changes, annular fissures, or internal disc disruption, leading to nociceptive input from the disc, and typically presents as predominant axial low back pain. However, the patient did not report predominant axial low back pain, and there was no interspinous tenderness on examination, making discogenic pain less likely [8].

#### 6) Myofascial pain syndrome

Myofascial or muscular pain arises from overuse, strain, or trigger points within the gluteal musculature, which can be reproduced by palpation or specific muscle activation. In this case, there was no focal tenderness over the gluteal musculature during the examination, and the severity of the patient’s functional limitation was greater than that expected from muscle pain alone [9].

### 2. Diagnosis and management

To determine whether lumbar radiculopathy contributed to the patient’s symptoms, a left L5 nerve root block with 1 mL of 2% lidocaine was performed [10]. The patient had no history of bleeding tendency or allergy to injectates. The nerve root block did not reduce the pain; therefore, lumbar radicular pain secondary to spinal stenosis at L4 to L5 was deemed less likely to be the cause of her left buttock pain.

**Fig. 2.**
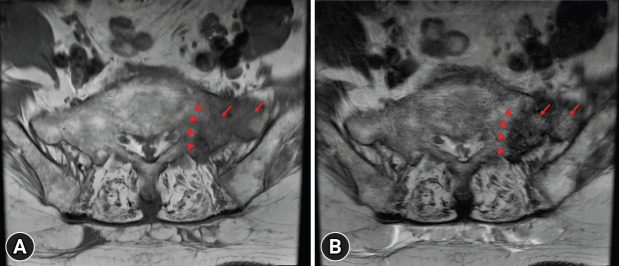
(A) Axial T1-weighted and (B) axial T2-weighted pelvic magnetic resonance imaging shows low T1 and high T2 signal intensity in the left sacral ala, indicative of bone marrow edema (arrows). Additionally, a hypointense fracture line (arrowheads) is visible laterally to the sacral foramina, consistent with sacral insufficiency fracture.

Given the positive Patrick’s FABER test and SIJ thrust test, which reproduced her left buttock pain, a pathology involving the left SIJ or sacral bone was considered. Therefore, pelvic MRI was performed as an additional diagnostic evaluation to assess SIJ or sacral bone pathology. Pelvic MRI demonstrated abnormal signal intensity in the left sacral ala, with low signal intensity on T1-weighted images and high signal intensity on T2-weighted images, suggesting bone marrow edema. In addition, a hypointense fracture line lateral to the sacral foramina was observed (Fig. 2). This finding was consistent with SIF, with osteoporosis as the likely underlying cause.

Considering the absence of neurological deficits and fracture displacement in imaging studies, conservative management was selected. We applied a lumbosacral orthosis and prescribed tapentadol (50 mg twice daily for 30 days), along with a transdermal buprenorphine patch (5 µg/hour, changed weekly). The restriction of activities that provoked pain was recommended.

## Clinical course

At the 3-month follow-up, after initiating conservative treatment, the patient’s left buttock pain had completely resolved.

## Discussion

In clinical practice, radicular pain due to spinal stenosis or disc herniation is often considered the most likely disorder when patients complain of buttock pain. In this case, lumbar spine MRI revealed central canal stenosis at the L4 to L5 level, and left L5 radicular pain was initially considered a plausible explanation for the severe left buttock pain and walking intolerance [2]. However, a diagnostic L5 nerve root block with lidocaine did not result in pain reduction, indicating that the pain was less likely to be attributable to lumbar spinal stenosis.

Attention then shifted to the possibility of SIJ-origin pain or pain from SIF given that the patient’s left buttock pain was reproduced by SIJ provocative maneuvers, and she experienced significant pain during ambulation. Pelvic MRI demonstrated findings consistent with SIF in the left sacral ala [4].

SIF is an osteoporotic stress fracture that most commonly affects the sacral alae. It usually presents in older adults with severe buttock or pelvic pain without major trauma. The symptoms of SIF can overlap with those of lumbar radicular or SIJ-origin pain and pelvic radiographs may be unremarkable. Pelvic MRI is a valuable diagnostic tool because it can detect sacral marrow edema and fracture-related changes. Most patients with SIF and without neurological deficits are treated conservatively with braces and analgesics, whereas sacroplasty may be considered for patients with persistent severe pain or mobility issues [4,5].

In conclusion, this case highlights the importance of considering SIF in older patients presenting with severe buttock or pelvic pain without antecedent trauma, particularly those with osteoporosis. Even in patients with coexisting lumbar or SIJ degenerative findings, SIF should remain a diagnostic consideration [4,5]. Early recognition of SIF and a timely pelvic MRI can prevent diagnostic delays and facilitate appropriate conservative management.
